# Radiolabeling of functional oligonucleotides for molecular imaging

**DOI:** 10.3389/fbioe.2022.986412

**Published:** 2022-08-19

**Authors:** Dunfang Liu, Qian Xia, Ding Ding, Weihong Tan

**Affiliations:** ^1^ Institute of Molecular Medicine (IMM), Renji Hospital, State Key Laboratory of Oncogenes and Related Genes, Shanghai Jiao Tong University School of Medicine and College of Chemistry and Chemical Engineering, Shanghai Jiao Tong University, Shanghai, China; ^2^ Department of Nuclear Medicine, Institute of Clinical Nuclear Medicine, Renji Hospital, School of Medicine, Shanghai Jiao Tong University, Shanghai, China; ^3^ The Cancer Hospital of the University of Chinese Academy of Sciences (Zhejiang Cancer Hospital), Hangzhou Institute of Medicine (HIM), Chinese Academy of Sciences, Hangzhou, Zhejiang, China; ^4^ Molecular Science and Biomedicine Laboratory (MBL), State Key Laboratory of Chemo/Biosensing and Chemometrics, College of Chemistry and Chemical Engineering, Hunan University, Changsha, Hunan, China

**Keywords:** functional oligonucleotides, radiolabeling, radiopharmaceutical, molecular imaging, precision medecine

## Abstract

Molecular imaging has greatly advanced basic biology and translational medicine through visualization and quantification of molecular events in a cellular context and living organisms. Nuclear medicine, including positron emission tomography (PET) and single-photon emission tomography (SPECT), is one of the most representative molecular imaging modalities which is widely used in clinical theranostics. Recently, numerous molecular imaging agents have been developed to improve the quality and expand the applicable diseases of molecular imaging. Based on the choice of specific imaging agents, molecular imaging is capable of studying tumor biological activities, detecting tumor metastasis, and imaging Alzheimer’s disease-related amyloid proteins. Among these imaging agents, functional oligonucleotides-based imaging probes are becoming increasingly important due to their unique features. Antisense oligonucleotides, small interfering RNA, and aptamers are privileged molecular tools in precision medicine for cancer diagnosis and treatment. These chemically synthesized oligonucleotides without batch-to-batch variations are flexible to incorporate with other molecules without affecting their functionalities. Therefore, through the combination of oligonucleotides with radioisotopes, a series of molecular imaging agents were developed in the past decades to achieve highly sensitive and accurate biomedical imaging modalities for clinical theranostic. Due to the nature of oligonucleotides, the strategies of oligonucleotide radiolabeling are different from conventional small molecular tracers, and the radiolabeling strategy with rational design is highly correlated to the imaging quality. In this review, we summarize recent advancements in functional oligonucleotide radiolabeling strategies and respective molecular imaging applications. Meanwhile, challenges and future development insights of functional oligonucleotide-based radiopharmaceuticals are discussed in the end.

## Introduction

Molecular imaging is an emerging technique that allows *in situ* visualization, characterization, and investigation of molecular biological processes in humans or other living systems (Massoud and Gambhir, 2003; Cherry, 2004). As the most representative clinical molecular imaging modalities, positron emission tomography (PET) and single-photon emission tomography (SPECT) realized noninvasive and real-time evaluation of disease-related biomarkers that laid a solid foundation not only in precision medicine but also in the study of biomarkers biological processes such as metabolism, hypoxia, proliferation, apoptosis, and angiogenesis (Luker and Piwnica-Worms, 2001; Pimlott and Sutherland, 2011; Derlin et al., 2018). Recently, taking advantage of targeting ligands such as antibodies and small molecules, receptors-overexpressed cells or tissues of lesions can be visualized and evaluated *via* PET imaging which facilitated the prediction of responses to targeted therapy and immune therapy (Wei et al., 2020). However, these targeting ligands suffered from several obstacles which hindered their further development. The large molecular weights (around 150 kD) of antibodies led to a prolonged blood half-life which adversely affected the target-to-background [T/B] ratio of imaging. Besides, ^18^F-fluorodeoxyglucose (^18^F-FDG) PET imaging is a kind of passive targeting that only reflect disease sites’ metabolism behaviors but the distribution of certain biomarkers.

To further improve the quality of molecular imaging, it is essential to develop novel imaging probes integrated with other functional molecular tools. Recent approaches demonstrated oligonucleotides possess fascinating biological functions beyond natural genetic materials. Through the precise hybridization process, antisense oligonucleotide (ASO) and small interfering RNA (siRNA) are capable of mediating cellular biological processes, and further achieving therapeutic purposes (Bennett, 2019; Hu et al., 2020). In addition, aptamer, which is a piece of DNA or RNA fragment, could interact with biomolecules or cell surface receptors specifically through the folding of conformation (Moutsiopoulou et al., 2019). Taking advantage of these unique features, these functional oligonucleotides were widely utilized in precision medicine for therapy, diagnosis, and imaging (Tavitian, 2003; Geary et al., 2015; Ding et al., 2020). Among these applications, novel radiopharmaceuticals based on functional oligonucleotide are of great interest due to the extraordinary advantages they provided. Oligonucleotides are fully chemical synthesized enabling readily large-scale production and strict quality control (Khvorova and Watts, 2017). Easy modification with a variety of chelators and precisely controlled conjugation sites make oligonucleotide a desirable radiolabeling platform for radiopharmaceutical developments with unlimited diversity. From the perspective of imaging, the general low MW of oligonucleotide facilitated fast clearance of the imaging probe once it accomplished imaging duty, greatly minimizing the radiation injuries to patients and the public.

**SCHEME 1:**
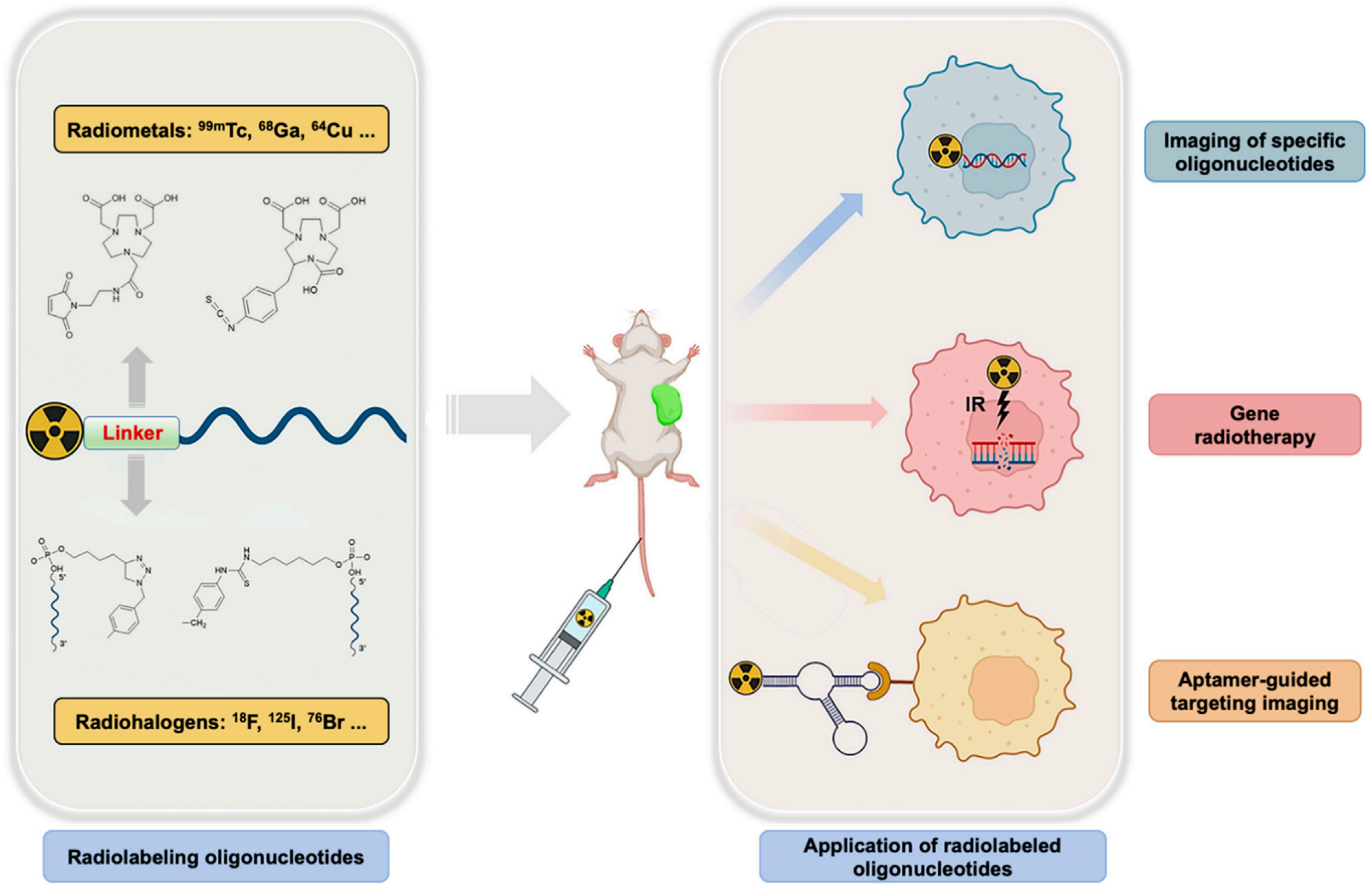
Radiolabeling of functional oligonucleotides and its molecular imaging applications.

To date, the majority of oligonucleotide-based molecular imaging applications could be briefly categorized into three categories and illustrated in scheme 1. 1) Imaging of specific oligonucleotides for the monitoring of ASO therapy (Bennett et al., 2019). ASO treatment relies on the complementary hybridization of an antisense oligonucleotide with a target oncogene mRNA sequence. Consequently, the radiolabeling antisense oligonucleotide is theoretically straightforward to realize noninvasive imaging of oncogene expression *in vivo* and predicts the responses of gene therapy. 2) Gene radiotherapy, which radiolabels oligonucleotides with therapeutic radionuclides, can effectively deliver considerable amounts of radionuclides into disease sites for therapy and reduce side effects on normal organs and tissues at the same time (Eckburg et al., 2020). 3) Aptamer-guided targeting imaging of disease-related biomarkers (Filippi et al., 2020). With the help of aptamers’ superior specific binding capacity, the combination of aptamers with radionuclides successfully realized targeted cancer theranostics (Song et al., 2022). To give a comprehensive landscape of functional oligonucleotides-based molecular imaging, herein, we review recently reported oligonucleotides radiolabeling strategies and highlight respective applications. Meanwhile, the future challenges and perspectives of oligonucleotides-based molecular imaging are also discussed in the end.

## Radiolabeling strategies of oligonucleotides

After decades of development, most strategies developed for antibodies, proteins, and peptides radiolabeling are well-established and can be applied to oligonucleotides directly. Moreover, chemically synthesized oligonucleotides provide diverse choices for the design of specific radiolabeling strategies. It is easy to introduce radiolabeling chelators or conjugation sites at any positions of oligonucleotides based on the requirements of radionuclides. In contrast to antibodies and proteins which may contain multiple potential labeling sites dispersed throughout their amino acid sequence, oligonucleotides can be radiolabeled at the predefined positions with precisely controlled amounts. Herein, we summarize recent oligonucleotide radiolabeling strategies and respective applications based on the species of labeled radionuclides.

## Radiometals

Transition metal radioisotope Technetium-99m (^99m^Tc, *T*_1/2_ = 6.02 h) is one of the most used radionuclides in SPECT diagnostics due to its splendid physical properties. Present radiolabeling strategies of the oligonucleotide with ^99m^Tc could be roughly divided into either direct or indirect methods. The directly radiolabeling of ^99m^Tc without any chelating agent seems to be more favorable because of the time and labor-saving labeling process. In 2014, the direct ^99m^Tc-labeling of oligonucleotide aptamers was reported by Correa et al. (Correa et al., 2014). Two oligonucleotide aptamers, with and without the primary amine group, were selected and radiolabeled with ^99m^TcO_4_^−^in the presence of stannous chloride as a reducing agent. Both oligonucleotide aptamers’ radiolabeling showed high radiochemical efficiencies and considerable *in vitro* stability and were further applied to carcinoembryonic antigen level evaluation. In addition, several direct radiolabeling approaches of oligonucleotide aptamers were reported by others with high radiolabeling efficiency (Charlton et al., 1997; dos Santos et al., 2015; de Sousa Lacerda et al., 2017). On the other hand, indirect ^99m^Tc labeling strategies of oligonucleotide relied on the chelators which were similar to antibodies and peptides (Hicke et al., 2006; Borbas et al., 2007; Varmira et al., 2014). Since ^99m^Tc is a metal isotope, the chelator molecule needs to be attached *via* a linker to avoid steric hindrances. The chelating agents such as Mercaptoacetyltriglycine (MAG3), Diethylenetriaminepentaacetic acid (DTPA), and Tetraazacyclododecane tetraacetic acid (DOTA), usually donates lone pairs of electrons to reduced ^99m^Tc to generate coordination bonds. In 2006, Wang et al. detailed reported ^99m^Tc radiolabeling strategies of biomolecules *via* MAG3 chelator (Wang et al., 2006). Besides, the cyclic anhydride DTPA has been widely used to radiolabel amine derivatized oligonucleotides with ^99m^Tc (Liu et al., 2009). Moreover, DOTA, which is a macrocyclic bifunctional chelating agent, binds to trivalent metals such as Indium (In^3+^), Yttrium (Y^3+^) rapidly under mild conditions and possesses better radiolabeling efficiencies in comparison with acyclic ligands, such as DTPA. It can be readily modified into a bifunctional chelating agent by the derivatization of one of the carboxylates (Schlesinger et al., 2009). In 2012, Ren et al. reported the ^99m^Tc radiolabeling of human tumor telomerase reverse transcriptase hTERT antisense oligonucleotide through a bifunctional chelator DOTA. In this approach, the ASO sequence was conjugated with DOTA-NHS at the 3′-end amine with more than 85% radiochemical purity (Ren et al., 2012).

Aside from ^99m^Tc, radiometals including ^64^Cu, ^68^Ga, ^111^In, and others were also frequently used for oligonucleotide radiolabeling (Table 1). Gallium-68 (^68^Ga, T_1/2_ = 1.1 h) is a highly promising positronium radionuclide that can be readily produced from ^68^Ge/^68^Ga generators. Typical ^68^Ga radiolabeling was realized through coordination chemistry between the Ga^3+^ and respective chelators such as DOTA. Recently, the Gallium (III) complex of (1,4,7-triazanonane-1,4,7-triyl) triacetic acid (NOTA) has been demonstrated thermodynamically more stable than DOTA, and radiolabeling *via* NOTA can be realized in room temperature under mild conditions with most biomolecules (Price and Orvig, 2014). For example, Gijs et al. successfully conjugated p-SCN-Bn-NOTA with aptamer and radiolabeled with ^68^Ga (Gijs et al., 2016). The main advantage of these bioconjugation approaches is that there is only one binding site for the chelator. Copper-64 (^64^Cu, T_1/2_ = 12.7 h) radiates both β^−^ decay and positron emission, produced by medical cyclotrons with highly specific activity (Bass et al., 2000; Anderson et al., 2008; Anderson and Ferdani, 2009). Because ^64^Cu undergoes β^−^ emission in addition to β^+^ emission, it is a promising theranostic radionuclide. In a recent study, Rockey et al. reported a ^64^Cu radiolabel oligonucleotide aptamer which binds to prostate specific membrane antigen (PSMA) specifically. Different chelators were investigated in this study including DOTA via the NHS ester, NOTA and PTCA *via* the 2-S-(4-Isothiocyanatobenzyl (p-SCN-Bn), and 1,8-Diamino-3,6,10,13,16,19-hexaazabicyclo [6,6,6]-eicosane (diAmSar) *via* a disuccinimidyl suberate (DSS) linker (Rockey et al., 2011). Besides, Li et al. reported the labeling of AS1411 aptamer with ^64^Cu for micro PET/CT study by conjugating four different chelators (Li et al., 2014). Nowadays, Zirconium-89 (^89^Zr, T_1/2_ = 78.4 h) has drawn increasing attention because of its fitting emission energy properties and long half-life. Due to its long half-life, it is currently used mainly for labelling biomolecules with long blood half-life, e.g., mAb. Desferrioxamine is the most used chelator for ^89^Zr radiolabeling which is usually conjugated to the biomolecule prior to the radiolabeling reaction (Meijs et al., 1992). The three hydroxamates and two additional anions or water molecules stabilize the Zr^4+^ ion through the generation of an octadentate structural complex in the desferrioxamine complex (Baroncelli and Grossi, 1965). Because of the oligonucleotide fast clearance nature in physiological milieu, ^89^Zr radiolabeled oligonucleotide without modification for PET imaging applications are still rare. Integrating oligonucleotides such as aptamers with nanomaterials or nanostructures might be able to take advantage of the aptamer’s targeting ability and mediate the blood half-life as well. In 2018, Fletcher et al. reported targeting functionalization of hyperbranched polymers with vascular endothelial growth factors (VEGF) targeted aptamer, and successfully applied for triple-negative breast cancer PET imaging (Fletcher et al., 2018).

**TABLE 1:** Characteristics of common nuclear medicine radionuclides and current methods of radiolabeling radioisotope to oligonucleotides.

Isotope	T_1/2_	Mode of decay (%)	Common production methods	Oligonucleotides functionalization	Radiolabeling strategy	Conjugation method	References
^99m^Tc	6 h	IT (γ89)	^99^Mo/^99m^Tc generator	5′ Hexylamine	Direct radiolabeling (TCEP as reducing agent)	None	Charlton et al. (1997)
				None or 5′ hexylamine	Direct radiolabeling (stannous chloride as reducing agent)	None	Correa et al. (2014)
				5′ Amine	Labeling *via* chelator (MetCyc, MAG3)	N-Acylation	Borbas et al. (2007)
				5′ Hexylamine	Labeling via chelator (HYNIC, EDDA)	N-Acylation	Varmira et al. (2014)
				3′ Amine	Labeling *via* chelator (DOTA)	N-Acylation	Ren et al. (2012)
^68^Ga	68.3 min	β^+^ (89)	^68^Ge/^68^Ga generator	5′ Dodecylamine	Labeling *via* chelator (DOTA, NOTA, PCTA, diAmSar)	N-Acylation	Gijs et al. (2016)
		EC (11)	^68^Zn (p,n)^68^Ga				
^64^Cu	12.7 h	β^+^ (17.9)	^64^Ni(p,n)^64^Cu	5′ Dodecylamine	Labeling *via* chelator (DOTA, NOTA, PCTA, diAmSar)	N-Acylation	Rockey et al. (2011)
		β^−^ (39) EC (43)	^64^Ni(d,2n)^64^Cu ^68^Zn (p,α)^64^Cu	3′ Amine	Labeling *via* chelator (DOTA, DOTA-Bn, NOTA-Bn, TE2A)	N-Acylation	Li et al. (2014)
^89^Zr	78.4 h	β^+^ (22.8)	^89^Y (p,n)^89^Zr	5′ DBCO	Labeling *via* chelator (DFO)	Alkyne-azide cycloaddition	Fletcher et al. (2018)
		EC (77)					
^90^Y	64.1 h	β^−^ (100)	^89^Y (n,γ)^90^Y	5′ Thiol	Labeling *via* chelator (DOTA)	S-Acylation	Schlesinger et al. (2009)
^177^Lu	6.7 d	β^−^ (100)	^176^ Yb(n,γ)^177^ Yb	5′ Hexylamine	Labeling via chelator (DTPA)	N-Acylation	Fathi et al. (2015)
^188^Re	16.9 h	β^−^ (72)	^188^W/^188^Re generator	3′Amine-	Labeling *via* chelator (MAG3)	N-Acylation	Liu et al. (2003)
		γ (15)					
^18^F	110 min	β^+^ (97)	^18^O (p,n)^18^F	5′ Hexylamine	Labeling *via* 4-([^18^F]Fluoromethyl) phenyl isothiocyanate	N-Alkylation	Hedberg and Léngstroma, (1997)
		EC (3)		5′ Thiol	Labeling *via* n-(4-[^18^F ]fluorobenzyl)-2-bromoacetamide, *a*-bromo-α'-[^18^F]fluoro-m-xylene	S-Alkylation	de Vries et al. (2003)
				5′ Alkyne	Labeling *via* ^18^F-fluorobenzyl azide	Alkyne-azide cycloaddition	Jacobson et al. (2015a)
				5′ Amine	Labeling *via* ^18^F-SFB	N-Alkylation	Jacobson et al. (2015b)
^125^I	59.5 d	EC (100)	^124^Xe (n,γ)^125^Xe	5′ Hexylamine	Labeling *via* PMPITC	N-Alkylation	Dewanjee et al. (1991)
				5′ Tyramine	Direct labeling	None	Cammilleri et al. (1996)
				5′ tributyl-stannylbenzamide	Direct labeling	None	Reed et al. (1997)
				5′ pyrimidine	Direct labeling	None	Takafuji et al. (2011)
^76^Br	16.2 h	β^+^ (57)	^76^Se (p,n)^76^Br	3′ Phosphoro-thioate monoester	Direct labeling	None	Kühnast et al. (2000)

Radiopharmaceutical therapy (RPT) delivers radioactive atoms to disease sites for therapeutic purposes, hosting several advantages over existing therapeutic approaches. Therapeutic radioisotopes such as Yttrium-90 (^90^Y, T_1/2_ = 64.1 h), Lutetium-177 (^177^Lu, T_1/2_ = 6.7 days), and Rhenium-188 (^188^Re, T_1/2_ = 16.9 h) were successfully incorporated with oligonucleotides for novel radiopharmaceutical developments as well. In 2009, Schlesinger et al. labeled l-RNAs with ^90^Y through DOTA-based maleimide reagents. Radiolabeling conditions and parameters were optimized in this approach (Schlesinger et al., 2009). ^177^Lu is a medium energy β^−^ emitter, which deposits its energy within a short range in tissues (maximum 2 mm). This property decreases the injuries to normal tissues and makes ^177^Lu well suited for the treatment of disseminated metastatic cancer (Das and Banerjee, 2016). Fathi et al. utilized fully phosphorothioate substitution and 2′-O-methoxy modified type 1 insulin-like growth factor receptor siRNA for ^177^Lu radiolabeling through p-SCN-Bn-DTPA chelator, and realized effective downregulation of the IGF-1R mRNA and proteins levels (Fathi et al., 2015). As another potential therapeutic radioisotope, ^188^Re can be utilized for theranostic which simultaneously realizes imaging and therapeutic goals. Liu et al. investigated a method of labeling oligonucleotide analog morpholino oligomers (MORFs) with ^188^Re for tumor pretargeting investigations (Liu et al., 2003). They found that the MAG3 chelator for post-conjugation labeling of morpholino hosting stable labeling with specific radioactivity was sufficiently high for radiotherapeutic applications without further purification.

## Radiohalogens

Fluorine-18 (^18^F, T_1/2_ = 110 min) is a well-known PET imaging radiopharmaceutical that possesses the most favorable physical properties for nuclear imaging. It is widely accepted that the development of ^18^F-FDG was the milestone of modern PET imaging history. As the most representative PET imaging agent, FDG acted a critical role in the field of cancer diagnostics such as the location of the primary tumor, and small metastasis. The possibility of incorporating ^18^F into functional oligonucleotide was first attempted by Hedberg and Léngstroma in 1997. In this study, one-step synthesis of radiolabeling precursor ([^18^F]fluoromethyl)phenyl isothiocyanate) was developed, and further ^18^F labeling of the oligonucleotide at 5′-end was achieved through a hexylamine linker (Hedberg and Léngstroma, 1997). Besides, oligonucleotides can be radiolabeled at 5′-end using [(^18^F)fluoromethyl]phenyl isothiocyanate. In 2003, de Vries et al. reported the radiolabeling of ASO using different alkylating agents and found n-[4-(^18^F)fluorobenzyl]-2-bromoacetamide and *a*-bromo -α'-[^18^F ]fluoro-m-xylene to be the most promising one with simple labeling procedures and high yields (de Vries et al., 2003). Alternatively, oligonucleotide can be radiolabeled with ^18^F through click chemistry. Radiolabeling of an SGC8 aptamer that targets PTK-7 protein receptor was realized using an alkynyl modified SGC8 aptamer and ^18^F-benzylazide *via* click chemistry (Jacobson et al., 2015a). The SGC8 aptamer was modified at the 5′-end with a terminal hexynyl group and the automated radiochemical synthesis of ^18^F-fluorobenzyl azide was achieved using an aromatic fluoride substitution on a spirocyclic hypervalent iodine (III) precursor. Further *in vivo* targeted PET imaging of tumor-bearing mice demonstrated the superiorities of aptamer-guided radiopharmaceuticals. In another approach, 2,5-dioxopyrrolidin-1-yl 4-(fluoro-^18^F) benzoate (^18^F-SFB) as a precursor of ^18^F was used to react with a primary amine-functionalized oligonucleotide. In 2015, Jacobson et al. reported ^18^F radiolabeling of a single-stranded DNA aptamer containing 70 nucleotides (Tenascin-C targeted aptamer) using ^18^F-SFB (Jacobson et al., 2015b). Radio-synthesis of ^18^F-SFB was performed in three automated steps using a 2-reaction-vial module (Jacobson et al., 2010), and the conjugation of ^18^F-SFB with the aptamer is carried out in basic sodium phosphate buffer, which results in low but usable yields. Additionally, aptamers or aptamer-containing oligonucleotide sequences can be hybridized to radiolabeled complementary sequences according to Watson and Crick base-pairing interactions. In 2016, Park et al. reported the development of the complementary oligonucleotide (cODN) hybridization-based aptamer conjugation approach for aptamer-based molecular imaging. The cODN was prelabeled with ^18^F and hybridized with a complementary sequence containing the AS1411 aptamer sequence in an aqueous buffer (Park et al., 2016).

Iodine-125 (^125^I, T_1/2_ = 59.5 days) is a radioisotope of iodine that is clinically ubiquitous in nuclear medicine imaging and brachytherapy for prostate cancer, uveal melanomas, and brain tumors. Because of its relatively long half-life and low-energy photons emission, ^125^I was also extensively used in the area of oligonucleotide-based radiotherapy and imaging. Conventional ^125^I radiolabeling is often accompanied by the use of prosthetic groups, Dewanjee et al. developed a new strategy of radioiodination by the conjugation of oligonucleotides with p-methoxyphenyl isothiocyanate (PMPITC) and optimized the radioiodination conditions. A 25-mer actin mRNA probe was modified by conjugation with the addition of aminohexyl (AH) group at the 5′-end and radioiodinated afterward for hybridization studies (Dewanjee et al., 1991). Besides, Cammilleri et al. developed a method allowing specific and stable radiolabeling of the ASO with ^125^I *via* a 5′-end grafted tyramine group. This transforming growth factor *a* targeted ASO probe provided information on the biodistribution of intratumoral administrated ASO (Cammilleri et al., 1996). In 1997, Reed et al. chemically modified oligonucleotide 5′-end with tributylstannylbenzamide and realized rapid and efficient radioiodination (Reed et al., 1997). This work developed a general and carrier-free strategy for oligonucleotides radioiodination. Recently, Takafuji et al. successfully radioiodinated a nucleolin-targeted AS1411 aptamer with ^125^I through the pyrimidine iodination in the presence of TlCl_3_ and realized *in vivo* tumor-targeted accumulation (Takafuji et al., 2011). As an artificially synthesized polymer oligonucleotide analog, peptide nucleic acid was radioiodinated by Adriana et al. (Tovar-Salazar et al., 2007). In this approach, a predesigned prosthetic group that incorporated both a radioiodinatable tyrosine and a triphenylphosphonium (TPP) moiety was synthesized, and the resultant ^125^I-labeled PNA probe was used for mRNA detection of the Lcn2 gene in Northern blotting. Bromine-76 (^76^Br, T_1/2_ = 16.2 h) is a positron-emitting isotope possessing a long half-life which allows long time organ kinetics studies. As part of the radiohalogens-based PET imaging, ^76^Br is also used to radiolabel oligonucleotide through noncarrier method (Kühnast et al., 2000).

## Perspective

Recent developments of oligonucleotide-based radiopharmaceuticals have proved the feasibility to be a potential imaging agent for targeted molecular imaging. Compared with conventional mAb targeting ligands, fully chemical synthesized oligonucleotide enables harsh labeling conditions which greatly extended the range of labeling radioisotopes and imaging modalities. The radiolabeling of functional oligonucleotides could be roughly categorized into either direct or indirect fashions, and each of them possesses its own merits and demerits as we summarized in Table 1. Basically, the choice of radiolabeling strategy is determined by the radionuclides. As represented by radiohalogans, radiolabeling of oligonucleotides was realized through a simple substitution reaction with low-cost and high reproducibility. Most importantly, in some special scenarios, the absence of chelators might be helpful to maintain the conformations of oligonucleotides which are highly associated with their functions. Indirect radiolabeling with chelators such as DOTA, DTPA is popular in radiometals. Radiolabeling oligonucleotides *via* chelators such as DOTA realized either ^68^Ga or ^177^Ru labeling that could be potentially utilized for cancer theranostic. In sum, rationally choosing radiolabeling strategies based on the demands of radionuclides is essential to the fabrication of functional oligonucleotides-based radiopharmaceuticals.

To date, functional oligonucleotide-based radiopharmaceuticals achieved a series of successes in the field of biomedicine. In the beginning, these radiolabeled probes were used for quantitative detection of diseases related biomarkers *in vitro*. Nowadays, along with the emergence of targeted PET imaging, functional oligonucleotides-based radiopharmaceuticals were widely used to achieve noninvasive evaluations of biomarkers *in vivo*, which is critical to precision medicine. It should be recognized that though functional oligonucleotide-based biomedical applications are promising, the clinic translation is still slow. Stability and specificity in the harsh physiological milieu largely hindered functional oligonucleotide further translation. Therefore, oligonucleotide-based molecular imaging should devote more efforts to the improvement of imaging agents’ *in vivo* specificity through chemical modifications or integration with nanomaterials.

In conclusion, this review summarizes recent progress of oligonucleotides-based radiopharmaceuticals in molecular imaging. A comprehensive of up-to-date oligonucleotides radiolabeling strategies is presented based on the type of radionuclides. Functional oligonucleotides-based molecular imaging applications including ASO, siRNA, and aptamer are highlighted. In the end, we make a perspective to discuss the challenges and opportunities of future oligonucleotides-based radiopharmaceuticals, and we hope this review could shed light on the future direction of functional oligonucleotides-based radiopharmaceuticals for both diagnostics and therapeutics.
